# Gut barrier dysfunction and the risk of ICU-acquired bacteremia- a case–control study

**DOI:** 10.1186/s13613-024-01280-8

**Published:** 2024-03-27

**Authors:** Meri R. J. Varkila, Diana M. Verboom, Lennie P. G. Derde, Tom van der Poll, Marc J. M. Bonten, Olaf L. Cremer, the MARS consortium, Friso M. de Beer, Lieuwe D. J. Bos, Gerie J. Glas, Arie J. Hoogendijk, Roosmarijn T. M. van Hooijdonk, Janneke Horn, Mischa A. Huson, Nicole P. Juffermans, Laura R. A. Schouten, Brendon Scicluna, Marcus J. Schultz, Marleen Straat, Lonneke A. van Vught, Luuk Wieske, Maryse A. Wiewel, Esther Witteveen, Marc J. M. Bonten, Olaf L. Cremer, Jos F. Frencken, Kirsten van de Groep, Peter M. C. Klein Klouwenberg, Maria E. Koster-Brouwer, David S. Y. Ong, Meri R. J. Varkila, Diana M. Verboom

**Affiliations:** 1 Julius Center for Health Sciences and Primary Care, University Medical Center Utrecht, Utrecht University, Utrecht, The Netherlands; 2 Department of Intensive Care Medicine, University Medical Center Utrecht, Utrecht, The Netherlands; 3 Center for Experimental and Molecular Medicine, Division of Infectious Diseases, Amsterdam University Medical Centers, University of Amsterdam, Amsterdam, The Netherlands; 4 Amsterdam Infection & Immunity Institute, Amsterdam University Medical Centers, Amsterdam, The Netherlands

**Keywords:** Bacteremia, Intensive care unit, Bloodstream infection, Bacterial translocation, Gut barrier, Gastrointestinal failure

## Abstract

**Background:**

Impaired intestinal barrier function can enable passage of enteric microorganisms into the bloodstream and lead to nosocomial bloodstream infections during critical illness. We aimed to determine the relative importance of gut translocation as a source for ICU-acquired enterococcal bacteremia of unknown origin.

**Methods:**

We conducted a nested case–control study in two mixed medical-surgical tertiary ICUs in the Netherlands among patients enrolled between 2011 and 2018. We selected 72 cases with ICU-acquired bacteremia due to enterococci (which are known gastrointestinal tract commensals) and 137 matched controls with bacteremia due to coagulase-negative staphylococci (CoNS) (which are of non-intestinal origin). We measured intestinal fatty acid-binding protein, trefoil factor-3, and citrulline 48 h before bacteremia onset. A composite measure for Gut Barrier Injury (GBI) was calculated as the sum of standardized z-scores for each biomarker plus a clinical gastrointestinal failure score.

**Results:**

No single biomarker yielded statistically significant differences between cases and controls. Median composite GBI was higher in cases than in controls (0.58, IQR − 0.36–1.69 vs. 0.32, IQR − 0.53–1.57, p = 0.33) and higher composite measures of GBI correlated with higher disease severity and ICU mortality (p < 0.001). In multivariable analysis, higher composite GBI was not significantly associated with increased occurrence of enterococcal bacteremia relative to CoNS bacteremia (adjusted OR 1.12 95% CI 0.93–1.34, p = 0.22).

**Conclusions:**

We could not demonstrate an association between biomarkers of gastrointestinal barrier dysfunction and an increased occurrence of bacteremia due to gut compared to skin flora during critical illness, suggesting against bacterial translocation as a major vector for acquisition of nosocomial bloodstream infections in the ICU.

**Supplementary Information:**

The online version contains supplementary material available at 10.1186/s13613-024-01280-8.

## Background

Critically ill patients are prone to nosocomial infection. Previous research reports that 18% of patients staying in the ICU for > 48 h develop new or recurrent infection (of any type) [1], whereas 6% acquire a new bloodstream infection (BSI) while in the ICU [2, 3]. Enteric microorganisms, such as enterococci, are frequent causes of these BSI events, and have been associated with an increased case fatality [4]. Although the etiology of ICU-acquired BSI is most commonly believed to be catheter-related [3, 4], some BSI events may be due to bacterial translocation from the gut.

Critical illness has a profound impact on gut integrity both by increasing apoptosis and decreasing proliferation of the intestinal epithelium, as well as by disrupting mucus integrity, which together results in increased gastrointestinal wall permeability. Bacterial translocation may ensue, allowing bacteria from the gut to enter the bloodstream, lymphatic fluids, and/or the peritoneal cavity [5–7]. Although this process may be clinically suspected in individual cases, it is difficult to determine the significance of intestinal barrier dysfunction and bacterial translocation in the etiology of ICU-acquired bloodstream infections at the population level. Gut integrity can, however, be assessed by measuring a number of circulating biomarkers, including intestinal fatty-acid binding protein (I-FABP), trefoil factor-3 (TFF3), and citrulline, which have all been demonstrated to correlate with mortality following gastrointestinal surgery and in cases of (suspected) bowel ischemia [8–14]. Based on these markers, it has previously been noted that enterocyte damage is quite common in ICU patients, occurring in up to half of a non-surgical ICU population [8].

Our study aims to determine the importance of gut barrier translocation (relative to other routes of contamination such as via the skin or the catheter hub) as a cause of ICU-acquired bacteremia in critically ill patients having no apparent origin for such infection. Quantitative knowledge about the most likely etiologic source of the cultured microorganism could prompt investigations into intestinal injury and/or ischemia, as well as aid clinicians in making informed decisions regarding catheter management and antibiotic treatment during episodes of ICU-acquired bacteremia.

## Methods

This study was performed using the Molecular Diagnosis and Risk Stratification of Sepsis (MARS) biorepository, for which consecutive patients had been prospectively enrolled in the mixed ICUs of two university medical centers in The Netherlands (AMC Amsterdam 2011–2013 and UMC Utrecht 2011–2018). The institutional review board of both study centers, Medical Research Ethics Committee Utrecht and Medical Research Ethics Committee AMC Amsterdam, approved an opt-out method of informed consent (IRB no. 10-1056C). Inclusion of patients has been described previously [15]. For the current analysis, we screened all patients with a length of stay > 2 days having at least a single BSI while in the ICU. An ICU-acquired bacteremia was subsequently defined as a first occurrence of a positive blood culture more than 48 h after ICU admission without a prior positive finding yielding the same pathogen for at least 30 days. Patients in whom bacteremia could be clinically attributed to a known source (e.g., secondary peritonitis due to perforation, abdominal abscess, deep surgical site infection, mediastinitis, endocarditis, etc.) were excluded. Further exclusion criteria included medical conditions that are known to impact the reliability of the measured biomarkers of gut dysfunction, such as chronic small bowel disease, chronic renal insufficiency, and abdominal surgery in the week prior to bacteremia occurrence.

We subsequently performed a matched case–control analysis among remaining subjects. Patients were classified as cases if they had ICU-acquired bacteremia due to *Enterococcus faecalis* or *Enterococcus faecium*, which are both commensal organisms of the human GI tract. All subjects having BSI with a pathogen that was of non-intestinal origin were potentially eligible as controls. However, as many microorganisms can occasionally colonize the intestine, we restricted the sampling of these control subjects to cases of bacteremia due to Coagulase-Negative Staphylococci (CoNS) only. Although CoNS can occasionally be cultured from the gut [16, 17], we assumed —for the purpose of a simplified analysis— that all observed CoNS bacteremia events had a non-intestinal origin, whereas we assumed that enterococcal bacteremia could arise from either the gut or from skin/catheter sources. Since enterococci are part of the normal human gut microbiota, whereas CoNS commonly live on the skin, and both species are of similar virulence [4], the relative frequency of BSI due to either one of these two species can be studied as a surrogate marker reflecting the proportional contributions of catheter- and gut-related routes of infection. All study subjects had indwelling intravascular catheters in place, and all selected positive blood culture episodes were followed by the start of empirical antibiotic therapy. However, to avoid misclassification of cases and controls, we adjudicated blood culture results based on a previously published algorithm [18]. This entailed excluding patients already receiving (empirical) antimicrobial therapy with activity against either enterococci or CoNS at time of blood culture draw and excluding patients in whom enterococci and CoNS had been cultured together (within a 48 h time window). Finally, to avoid including bacteremia episodes that in fact represented contaminated blood draws, only positive blood cultures having an incubation time shorter than 3 days were eligible for selection.

For each case, two control subjects were randomly selected using incidence density sampling. Matching criteria were age (± 10 years), length of stay in ICU at bacteremia onset (± 3 days), and the presence of acute kidney injury (defined as RIFLE stage ≥ 2 (injury)) in the 48 h prior to blood culture collection, since these factors may act as either confounders or effect-modifiers of the association between gut failure and BSI [8, 14]. Subjects were additionally matched on their year of presentation (± 4 years) in order to mitigate any possible effects of differences in plasma storage time.

### Biomarkers of gastrointestinal integrity

To quantify gut barrier integrity, we measured three circulating biomarkers of enterocyte damage and/or dysfunction in plasma as well as a clinical gastrointestinal failure score (Table 1). For this, we used samples and data that had been collected during the 48 h time period immediately preceding the bacteremia event. This time period was based on the observed biomarker kinetics in a pilot study that we had conducted in 20 patients with enterococcal bacteremia (Additional file 1: Fig S1). All biomarkers were measured in leftover EDTA plasma collected during routine care. These samples had been spun at 1500 rpm for 15 min and were stored at − 80 °C within 4 h (median) of blood draw. IFABP and TFF3 concentrations were measured using microfluidic single plex immunoassays (Ella system, Protein Simple, Bio-Techne, Abingdon, UK). Citrulline was measured using an ultra-performance liquid chromatography-electrospray tandem mass spectrometry assay (Xevo TQ MS, Waters, Milford, US). Threshold values for the biomarkers were defined based on previous literature as follows: elevated I-FABP > 100 pg/ml, elevated TFF3 > 8.3 ng/ml, and decreased citrulline < 20 μmol/l [8, 11, 19]. Finally, to quantify clinical aspects of gut dysfunction we used Reintam’s gastrointestinal failure score, which had been prospectively recorded [20]. This score is a 5-range scale based on clinically observed gastrointestinal symptoms and intra-abdominal pressure and ranges from 0 to 4, with higher scores indicating a higher degree of gastrointestinal dysfunction.

**Table 1:** Biomarkers of gastrointestinal barrier integrity

Source	Marker	Clinical relevance	Behavior in Gut Injury
Plasma	I-FABP	• Marker of enterocyte damage • Released in enterocyte membrane rupture and enterocyte necrosis	↑
Plasma	Citrulline	• Marker of functional enterocyte mass • High positive relationship with functional length of small bowel	↓
Plasma	TFF3	• Secreted mainly by goblet cells throughout small and large intestine • Important role in gastrointestinal defence and repair, as well as in the maintenance of the integrity of the intestinal barrier • Rapidly up-regulated at the margins of mucosal injury → reflective of intestinal epithelial damage	↑
Clinical records	GIF-score	• Clinical score: 0- Normal gastrointestinal function 1- Enteral feeding < 50% of calculated needs or no feeding 3 days after abdominal surgery 2- Food intolerance or intra-abdominal hypertension 3- Food intolerance and IAH 4- Abdominal compartment syndrome	↑

### Statistical analysis

To allow modelling of gut barrier dysfunction as a single exposure factor during data analysis, further parameterization of the biomarkers was necessary. To this end, we a priori constructed a composite measure of Gut Barrier Integrity (GBI). First, the measured concentrations for I-FABP, TFF3 and citrulline were log-transformed. These individual biomarkers were then expressed as standardized z-scores, calculated based on the observed distributions our study population. Because cases and controls were not equally represented in our study sample (due to 1:2 matching) we temporarily constructed a weighed pseudo-population for this. Finally, composite GBI was calculated as the sum of the three z-scores for the individual plasma biomarkers plus the (unprocessed) gastrointestinal failure score value.

We used multivariable unconditional logistic regression analysis to assess the association between gut barrier dysfunction (as measured by composite GBI) and the occurrence of ICU-acquired bacteremia with gut commensals (as indicated by the case–control status). In this analysis we used age and renal replacement therapy (RRT) as covariates to address any residual confounding that was not fully accounted for by the matching procedure. We also performed stratified analyses based on the presence of acute kidney injury and/or use of RRT, severity of disease (as measured by the Sequential Organ Failure Assessment (SOFA) score), and ICU mortality.

## Results

Among 6,916 eligible ICU admissions (73,144 observation days) available in the MARS biorepository we identified 717 ICU-acquired BSI episodes due to enterococci or CoNS, yielding an incidence rate of 3.8 (95% CI 3.4–4.2) and 7.6 (95% CI 7.0–8.2) events per 1000 observation days at risk, respectively. Following exclusion criteria, 444 bacteremia events remained eligible for study inclusion (Additional file 1: Fig S2). Among these, 72 (88%) of 82 cases of ICU-acquired enterococcal bacteremia could be matched to 137 controls with CoNS bacteremia. Patient demographics, comorbidities and characteristics of the ICU stay were comparable between cases and controls (Table 2). However, despite well balanced disease severity indicators between groups and similar total length of stay, patients with enterococcal bacteremia had higher ICU mortality (46% versus 31%, p = 0.03).

**Table 2:** Patient Characteristics

Variables	Cases		Controls		*P* Value
	N = 72		N = 137		
Patient characteristics at baseline					
Age (years)	63	(51–70)	63	(52–69)	*MF*
Male	50	(69.4)	94	(68.6)	0.90
Charlson comorbidity index	3	(2–4)	3	(1–4)	0.25
Immunodeficiency	16	(22.2)	25	(18.3)	0.50
Mucositis or intestinal GvHD	5	(6.9)	4	(2.9)	0.17
Previously admitted to ICU	12	(16.7)	18	(13.1)	0.49
Surgical reason for admission	29	(40.3)	47	(34.3)	0.39
Sepsis at admission	22	(30.6)	31	(22.6)	0.21
APACHE IV score	88.5	(65–104)	86	(68–102)	0.94
ICU outcomes					
Total length of ICU stay (days)	16	(9.5–26)	16	(10–26)	0.81
ICU mortality	33	(45.8)	42	(30.7)	*0.03*
Patient characteristics at bacteremia onset^a^					
Days in ICU	7	(5–12.5)	8	(5–12)	*MF*
SOFA score	7	(4.5–12.5)	7	(4–11)	0.14
Acute kidney injury	28	(38.9)	51	(37.2)	*MF*
Catecholamine use	39	(54.2)	68	(49.6)	0.53
C-reactive protein (mg/L)	107	(63–222)	112	(64–182)	0.80
Indwelling catheters					
Arterial catheter	68	(94.4)	133	(97.1)	0.35
CVC	59	(81.9)	100	(73.0)	0.15
Pulmonary catheter	1	(1.4)	9	(6.6)	0.10
RRT	24	(33.3)	38	(27.7)	*MF*
Plasma biomarkers					
I-FABP (pg/ml)	1056	(669–1820)	947	(545–1666)	0.21
TFF3 (ng/ml)	27.0	(12.4–47.9)	21.3	(11.3–43.4)	0.38
Citrulline (µmol/L)	25.8	(19.4–34.1)	25.3	(17.6–34.5)	0.84
GIF-score^a,b^ ≥ 2	20	(27.8)	32	(23.4)	0.59

Data are presented in median (interquartile range) or in absolute number (percentage)

*MF* matching factor, *GvHD* graft versus host disease, *APACHE* Acute Physiology And Chronic Health Evaluation, *SOFA* Sequential Organ Failure Assessment, *CVC* central venous catheter; *RRT*, renal replacement therapy, *I-FABP* intestinal fatty acid-binding protein, *TFF3* trefoil factor-3, *GIF* gastrointestinal failure

^a^As present (or measured) in the 48 h time window immediately preceding the blood culture draw that (later) yielded a first positive result

^b^GIF score: 0 = normal gastrointestinal function; 1 = enteral feeding under 50% of calculated needs or no feeding 3 days after abdominal surgery; 2 = food intolerance (FI) or intra-abdominal hypertension (IAH); 3 = FI and IAH; and 4 = abdominal compartment syndrome

**Fig. 1:**
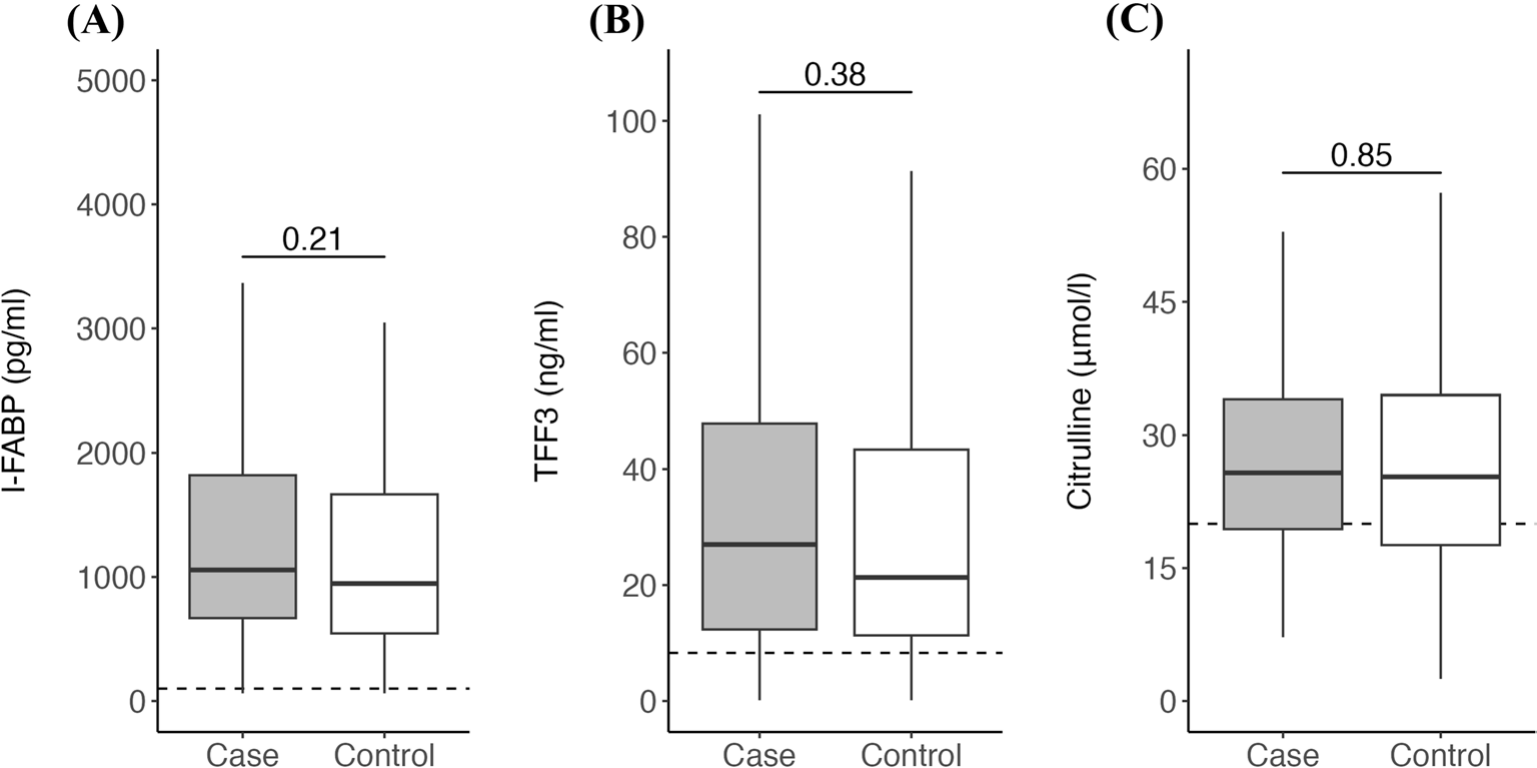
Plasma biomarkers of gut injury in cases and controls. Blood was drawn from patients in the 48 h preceding a bacteremia event. Plasma levels of (**A**) I-FABP, (**B**) TFF3 and (**C**) citrulline did not significantly differ between patients with enterococcal bacteremia as compared to patients with CoNS bacteremia. Dashed lines represent threshold values for biomarkers: elevated I-FABP > 100 pg/ml, elevated TFF3 > 8.3 ng/ml, and decreased citrulline < 20 μmol/l. *I-FABP* intestinal fatty acid-binding protein, *TFF3* trefoil factor-3, *CoNS* Coagulase-negative Staphylococci

Figure 1 shows the results for the individual plasma biomarkers of gastrointestinal barrier integrity. In our study population, I-FABP > 100 pg/ml (i.e., above the reported threshold associated with mucosal damage [8]) was observed during 201 (96%) of 209 bacteremia events, suggesting almost universal presence of (some) enterocyte destruction. Similarly, TFF3 > 8.3 ng/ml (i.e., above the 1.5 interquartile threshold observed in healthy volunteers [11]) was present during 182 (87%) events. However, we observed no relevant differences in either of these markers between cases and controls. In contrast, citrulline concentration > 20 μmol/l (i.e., within the normal range [19]) was mostly present, although very low levels (< 10.0 μmol/l) were observed during 9 (4%) BSI episodes. Yet once more, citrulline concentrations were similar between groups. Finally, GIF score ≥ 2 was observed during 52 (25%) events, indicating a high prevalence of gastrointestinal dysfunction and food intolerance in our study cohort, again with scores being comparable between cases and controls.

**Fig. 2:**
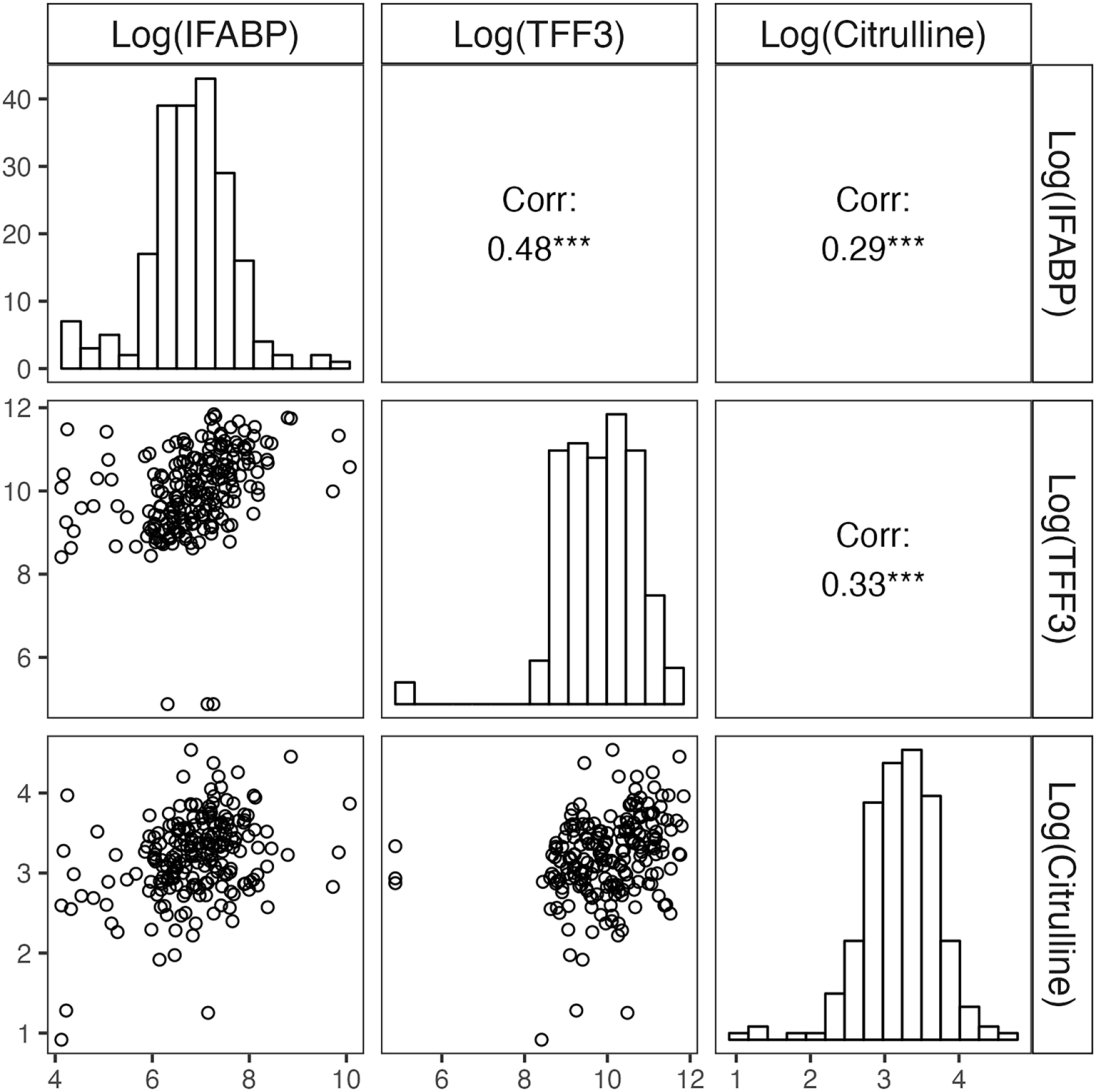
Correlations between plasma biomarkers. Plasma levels of I-FABP were moderately positively correlated with plasma levels of TFF3 (Spearman rho, r_s_ = 0.48, p < 0.001), while I-FABP and citrulline and TFF3 and citrulline were weakly positively correlated (r_s_ = 0.29, p < 0.001 and r_s_ = 0.33, p < 0.001, respectively). Histograms represent the distributions of plasma levels of I-FABP, citrulline and TFF3 after log-transformation. Scatterplots represent the correlations of the biomarker plasma levels after log-transformation. *I-FABP* intestinal fatty acid-binding protein, *TFF3* trefoil factor-3

Whereas I-FABP and TFF3 levels were moderately correlated (Spearman rho, r_s_ = 0.48, p < 0.001), we only observed weak correlations between I-FABP and citrulline and between TFF3 and citrulline (r_s_ = 0.29, p < 0.001 and r_s_ = 0.33, p < 0.001, respectively) (Fig. 2). Furthermore, these correlations were positive (where a negative relation had been expected). We also did not observe clear correlations between GIF score and plasma biomarker levels (Additional file 1: Fig S3).

After standardization as Z scores, the three biomarker measurements and clinical gastrointestinal failure score were combined into a single composite measure of GBI. Median composite GBI was 0.58 (IQR − 0.36–1.69) and 0.32 (IQR − 0.53–1.57) for patients with enterococcal and CoNS bacteremia, respectively (p = 0.33). In logistic regression analysis higher composite GBI was not associated with an increased occurrence of enterococcal bacteremia relative to CoNS bacteremia (crude OR 1.10, 95% CI 0.93–1.30, p = 0.27; adjusted OR 1.12 95% CI 0.93–1.34, p = 0.22).

Pre-specified analyses with stratification of patients according to the presence of acute kidney injury, severity of disease, and ICU mortality, did not demonstrate any significant associations (Additional file 1: Fig S4). However, all stratified analyses revealed a trend towards higher composite GBI in the enterococcal BSI cases compared to CoNS controls (adjusted OR for patients with high SOFA scores 1.23, 95% CI 0.95–1.60, p 0.12; for ICU non-survivors 1.24, 95% CI 0.91–1.72, p 0.18; for patients with acute kidney injury 1.27 95% CI 0.93–1.76, p 0.14) (Table 3). This suggests a possible role for gut barrier translocation in enterococcal BSI in the most severely ill patients having multi-organ failure. It must be noted, however, that the case–control matching was broken for these stratified analyses (yielding possible confounding).

**Table 3:** Multivariable logistic regression analyses of the association between composite Gut Barrier Injury and ICU bacteremia

Analysis	Cases (n)	Controls (n)	OR	(95% CI)	p
Primary analysis	72	137	1.12	(0.93–1.34)	0.22
Stratified analyses					
Acute kidney injury^a^	28	51	1.27	(0.93–1.76)	0.14
High severity of disease^b^	44	73	1.23	(0.95–1.60)	0.12
ICU non-survivors	33	42	1.24	(0.91–1.72)	0.18

All analyses were adjusted for age, and renal replacement therapy

*OR* odds ratio, *CI* confidence interval, *ICU* Intensive Care Unit

^a^Risk, Injury, Failure, Loss of kidney function, and End-stage kidney disease (RIFLE)-score of 2 or higher

^b^Sequential Organ Failure Assessment (SOFA)-score of 7 or higher

## Discussion

We measured known biomarkers of intestinal function and integrity in carefully matched patients with ICU-acquired bacteremia caused by pathogens of either certain intestinal of non-intestinal origin, and observed only minute differences between both groups. Although high I-FABP and TFF3 levels suggested that gut barrier integrity was compromised in a large proportion of our study population, we did not find an association with the preferred occurrence of ICU-acquired bacteremia caused by gut compared to skin flora. Furthermore, citrulline levels were mostly in the normal range and Reintam’s score indicated that clinically overt gut barrier dysfunction was rare. Together these observations argue against translocation from the intestine as a major source of bacteremia in critically ill patients.

Our findings contradict previous reports that have suggested a clinically relevant association between gut barrier dysfunction and the occurrence of bacteremia [21–25]. However, evidence of this relationship has been largely circumstantial and limited to surgical patients undergoing laparotomy only. Three studies, including a total of 1330 subjects undergoing laparotomy, cultured bacteria from intraoperative samples of mesenteric lymph nodes in 10–21% of patients [23–25]. While postoperative infectious complications were approximately twice as frequent in these patients, bacteremia events remained rare (or underreported). Of note, concordance between the intraoperative translocation specimen and blood culture was low and none of the studies performed multivariable analysis to adjust for confounding by other underlying pathology or infectious foci. Furthermore, our findings are in accordance with a study that has attempted to systematically assess the occurrence of bacterial translocation to the bloodstream during critical illness by directly sampling portal vein blood in trauma patients, observing that whereas 6 (30%) of 20 subjects developed multiple organ failure, only 2% of blood cultures became positive [26].

As gut permeability cannot be observed directly nor defined precisely, we attempted to capture this concept by measuring four known plasma biomarkers of intestinal barrier function and integrity before bacteremia onset. Because no generally accepted thresholds for these biomarkers existed, we standardized the biomarker measurements as z-scores. We then employed a composite score to measure the relative degree of intestinal injury. I-FABP is a cytosolic protein abundant in enterocytes along the entire small intestine and is normally not detectable in plasma [27–29]. When intestinal mucosal damage occurs, I-FABP is released into the circulation and its plasma concentration increases. I-FABP plasma levels > 100 pg/ml have been associated with enterocyte destruction during low-flow states associated with mesenteric ischemia [9, 10, 30]. TFF3 is a small peptide predominantly secreted by goblet cells of both the small and large intestine. Its expression is upregulated in response to mucosal damage and serves to promote mucosal barrier repair and inhibit enterocyte apoptosis. Elevated TFF3 has been associated with shock and multiple organ damage in critically illness [11, 31]. Citrulline is an amino acid that is synthesized from glutamine and arginine by mature enterocytes and its concentration in plasma therefore reflects functional enterocyte mass. Citrulline levels are correlated strongly with small bowel length and disease activity in various (chronic) enteropathies, and conversely to I-FABP and TFF-3, are known to decrease in critical illness and sepsis [12, 32]. By combining these markers into a composite measure, we were able to capture and quantify a range of gastrointestinal pathophysiology in a single parameter.

The selection of biomarkers for this study was based on the premise that loss of enterocyte integrity is the main driver of gut barrier failure and ensuing bacterial translocation. However, it is also possible that other mechanisms, such increased paracellular permeability, breakdown of the mucus layer, or decreased mucosal immunity, play a role in the translocation of gut organisms into the bloodstream [8]. Contrary to our a priori expectations, observed plasma citrulline levels were mostly normal in our study population. Furthermore, we observed a weak positive correlation between citrulline and TFF-3 and I-FABP concentrations, where a negative correlation was anticipated. This questions whether biomarker levels in our study were reflecting intestinal barrier function as intended. Indeed, correct interpretation of plasma citrulline concentrations in critically ill patients may be difficult due to its complex metabolism [14]. Previous research suggests ICU interventions, such as enteral nutrition or catecholamine use, can have compounded effects on the gut mucosa and biomarker kinetics [33, 34]. Additionally, sepsis of both digestive and extradigestive origin has been associated with decreased citrulline levels [32]. While stratified analyses of biomarker levels in our study did not indicate that differences between cases and controls were concealed by measurement error due to impaired renal clearance or severity of disease, our study design did not allow for in-depth investigation of biomarker dynamics.

Despite the overall negative study findings, our analyses do not exclude an etiologic link between loss of gut barrier integrity and the occurrence of bacteremia with enteric micro-organisms in some ICU patients. In fact, trend effects observed in our subgroup analyses suggest that this could be the case for the most severely ill individuals, particularly those with shock and/or sepsis. Indeed, acute mesenteric ischemia has been described as one of the leading causes of enterocyte damage in the critically ill [8], and sepsis is known to reduce enterocyte proliferation in crypts and inhibit epithelial migration along villi [8, 21], as well as induce apoptosis of enterocytes [35–37]. It is therefore quite reasonable to assume that these patients will be at high risk of bacterial translocation, even though our data did not demonstrate this. The role of bacterial translocation in subgroups of patients with established intestinal failure or mucosal injury should be further elucidated. Furthermore, several studies in patients with the acute respiratory distress syndrome have demonstrated enrichment of the lung microbiome with gut-flora in the absence of documented bacteremia [5, 38, 39], suggesting that bacterial translocation may be only a brief and transient event that is not always captured by blood cultures. Future studies should address whether gut barrier failure precedes or follows onset of severe critical illness, and how this relates to the timing of bacteremia events and the development of various extraintestinal pathobiomes.

Our study has some limitations. Although CoNS are generally considered non-enteric organisms, they may occasionally colonize the gut [16, 17], and CoNS infections of intestinal origin have indeed been described in neonates and cancer patients with mucositis [40]. If gut colonization occurred in a substantial proportion of control subjects, any existing differences between cases and controls could have been negated (thus obscuring a true association between translocation and enterococcal bacteremia). However, we find it unlikely that this played a significant role in our adult study population with a low prevalence of mucositis. Second, as our study focused exclusively on ICU-acquired bacteremia (occurring after at least 48 h after admission) with biomarker measurements that were performed median 7 days after ICU admission, we cannot draw conclusions related to occurrence of bacterial translocation during more acute stages of critical illness or directly compare our results to studies where enterocyte biomarkers were measured at earlier time points. Third, even though adjudication of blood culture results was based on a previously published algorithm that included concomitant culture characteristics, time-to-positivity, and prior antimicrobial therapy [18], some BSI events selected for this study may have represented contaminated blood draws rather than true bacteremia events. Fourth, we devised a non-validated composite measure to assess gut integrity, whereas use of the recently published Gastrointestinal Dysfunction Score (GIDS) or additional biomarkers for gastrointestinal functioning or mucosal immunity could have improved the robustness of our analysis [6, 41]. Finally, paracellular permeability and mucosal integrity are important components of gut barrier function that can be evaluated directly using methods, such as electron microscopy of intestinal tissue, or measuring the urinary excretion of orally administered tracer molecules or nondigestible sugars like lactulose and mannitol [6]. Lamentably these investigations could not be performed in this retrospective study.

## Conclusions

We could not demonstrate an association between a panel of biomarkers reflecting gastrointestinal injury and the preferred occurrence of bacteremia due to gut compared to skin commensals. This suggests that bacterial translocation is an infrequent mode of acquisition of nosocomial bloodstream infections in critically ill patients. Furthermore, these biomarkers are unlikely to be of much diagnostic use to clinicians for making informed decisions regarding the initiation of antibiotic therapy and/or catheter management following episodes of bacteremia caused by enterococci and other enteric bacteria in the ICU.

### Supplementary Information

**Additional file 1: Fig S1.** Pilot analysis of biomarker levels in days preceding blood culture. A) I-FABP and B) citrulline levels were measured in 18 patients with enterococcal bacteremia on the three days preceding (days -3 to -1), as well as on the day of blood culture draw (day 0). Biomarker levels remained relatively stable over the course of this time period. Abbreviations: I-FABP, intestinal fatty acid-binding protein. **Fig S2.** Flowchart. **Fig S3.** Plasma biomarkers and level of clinical GIF-score. Blood was drawn from patients in the 48 hours preceding a bacteremia event. Plasma levels of (i) I-FABP, ii) TFF3 and iii) citrulline did not significantly differ between patients with Gastrointestinal Failure score 0, 1 or 2 or higher. Abbreviations: I-FABP, intestinal fatty acid-binding protein; TFF3, trefoil factor-3. Fig 4. Plasma biomarkers within subgroups from stratified analyses. Blood was drawn from patients in the 48 hours preceding a bacteremia event. In stratified analyses for A) presence of acute kidney injury, B) severity of disease or C) ICU mortality, plasma levels of (i) I-FABP, ii) TFF3 and iii) citrulline did not significantly differ between patients with enterococcal bacteremia as compared to patients with CoNS bacteremia. Acute kidney injury was defined as a RIFLE-score of 2 or higher as present (or measured) in the 48-hour time window immediately preceding the blood culture draw that (later) yielded a first positive result. High severity of disease was defined as Sequential Organ Failure Assessment- score of 7 or higher. Abbreviations: I-FABP, intestinal fatty acid-binding protein; TFF3, trefoil factor-3.

## Data Availability

Data privacy prohibits deposition of individual level data to public repositories and the ethical approval does not cover public sharing of data for unknown purposes. Upon contact with the authors an institutional data transfer agreement may be established, and data shared if the aims of data use are covered by ethical approval.

## Abbreviations

APACHE: Acute physiology and chronic health evaluation
BSI: Bloodstream infection
CoNS: Coagulase-negative staphylococci
CVC: Central venous catheter
GBI: Gut barrier injury
GIDS: Gastrointestinal dysfunction score
GIF: Gastrointestinal failure
ICU: Intensive Care Unit
I-FABP: Intestinal fatty acid-binding protein
MARS: Molecular diagnosis and risk stratification of sepsis cohort
RRT: Renal replacement therapy
SOFA: Sequential organ failure assessment
TFF3: Trefoil factor-3
