# Supplementary material for: Gut barrier dysfunction and the risk of ICU-acquired bacteremia- a case–control study
Source: Ann Intensive Care. 2024 Mar 27;14:42. doi: 10.1186/s13613-024-01280-8 (PMC10973289; doi:10.1186/s13613-024-01280-8)
Supplement: Supplementary file 1 — Additional file 1: Fig S1. Pilot analysis of biomarker levels in days preceding blood culture. A) I-FABP and B) citrulline levels were measured in 18 patients with enterococcal bacteremia on the three days preceding (days -3 to -1), as well as on the day of blood culture draw (day 0). Biomarker levels remained relatively stable over the course of this time period. Abbreviations: I-FABP, intestinal fatty acid-binding protein. Fig S2. Flowchart. Fig S3. Plasma biomarkers and level of clinical GIF-score. Blood was drawn from patients in the 48 hours preceding a bacteremia event. Plasma levels of (i) I-FABP, ii) TFF3 and iii) citrulline did not significantly differ between patients with Gastrointestinal Failure score 0, 1 or 2 or higher. Abbreviations: I-FABP, intestinal fatty acid-binding protein; TFF3, trefoil factor-3. Fig 4. Plasma biomarkers within subgroups from stratified analyses. Blood was drawn from patients in the 48 hours preceding a bacteremia event. In stratified analyses for A) presence of acute kidney injury, B) severity of disease or C) ICU mortality, plasma levels of (i) I-FABP, ii) TFF3 and iii) citrulline did not significantly differ between patients with enterococcal bacteremia as compared to patients with CoNS bacteremia. Acute kidney injury was defined as a RIFLE-score of 2 or higher as present (or measured) in the 48-hour time window immediately preceding the blood culture draw that (later) yielded a first positive result. High severity of disease was defined as Sequential Organ Failure Assessment- score of 7 or higher. Abbreviations: I-FABP, intestinal fatty acid-binding protein; TFF3, trefoil factor-3. [file 13613_2024_1280_MOESM1_ESM.docx]

**ADDITIONAL FILES**

Gut barrier dysfunction and the risk of ICU-acquired bacteremia- a case-control study

Meri RJ Varkila, Diana M Verboom, Lennie PG Derde, Tom van der Poll, Marc JM Bonten, Olaf L Cremer; on behalf of the MARS consortium

Contents:

**Additional Fig 1.** Pilot analysis of biomarker levels in days preceding blood culture

**Additional Fig 2.** Flowchart

**Additional Fig 3.** Plasma biomarkers and level of clinical GIF-score

**Additional Fig 4.** Plasma biomarkers within subgroups from stratified analyses

**Additional Fig 1. Pilot analysis of biomarker levels in days preceding blood culture**


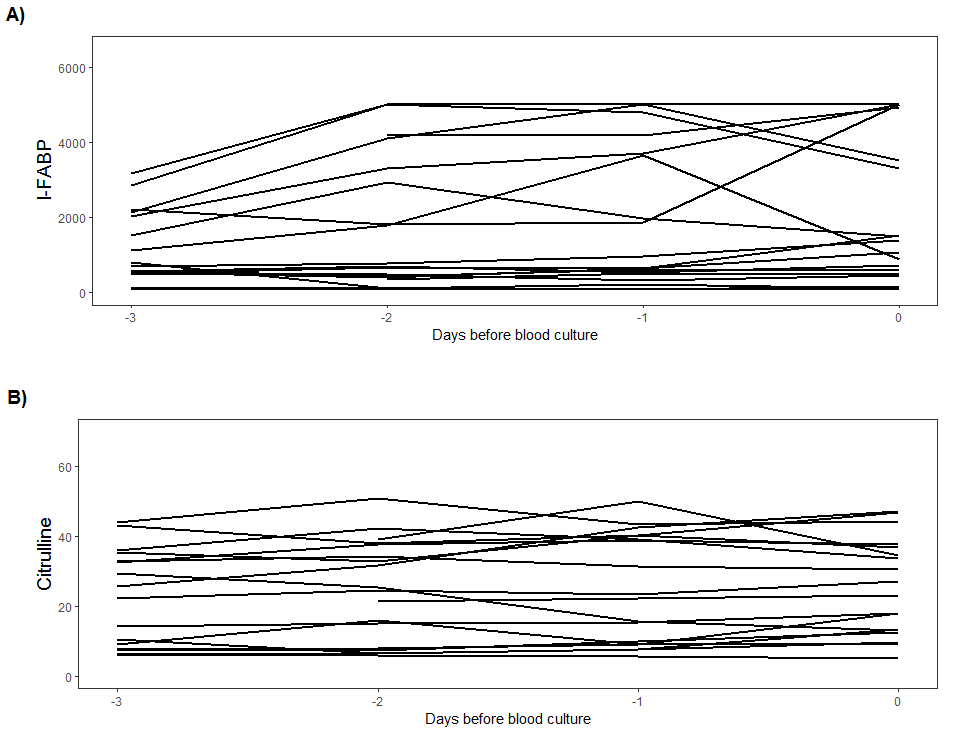


A) I-FABP and B) citrulline levels were measured in 18 patients with enterococcal bacteremia on the three days preceding (days -3 to -1) , as well as on the day of blood culture draw (day 0). Biomarker levels remained relatively stable over the course of this time period. Abbreviations: I-FABP, intestinal fatty acid-binding protein.

**Additional Fig 2. Flowchart**


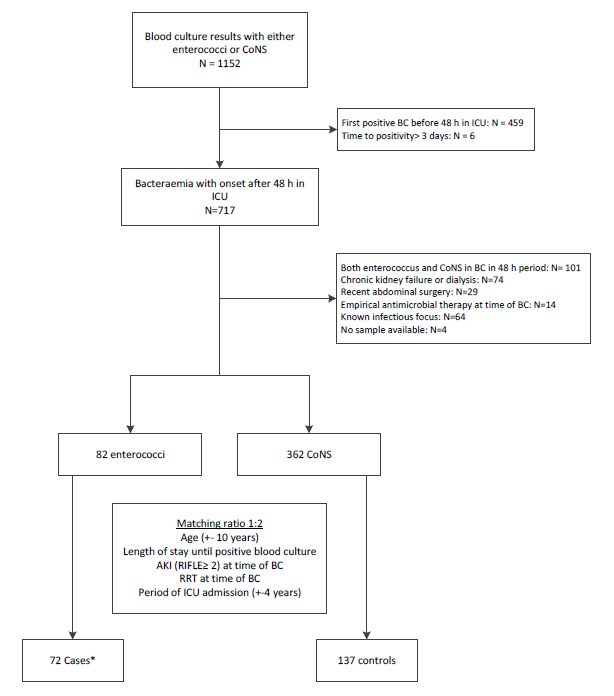


*Among these, 7 individuals were matched with 1 control.

Abbreviations: CoNS, Coagulase-negative Staphylococci; BC, blood culture; ICU, Intensive Care Unit; AKI, Acute Kindey Injury; RIFLE-score, Risk, Injury, Failure, Loss of kidney function, and End-stage kidney disease-score; RRT, renal replacement therapy.

**Additional Fig 3. Plasma biomarkers and level of clinical GIF-score**


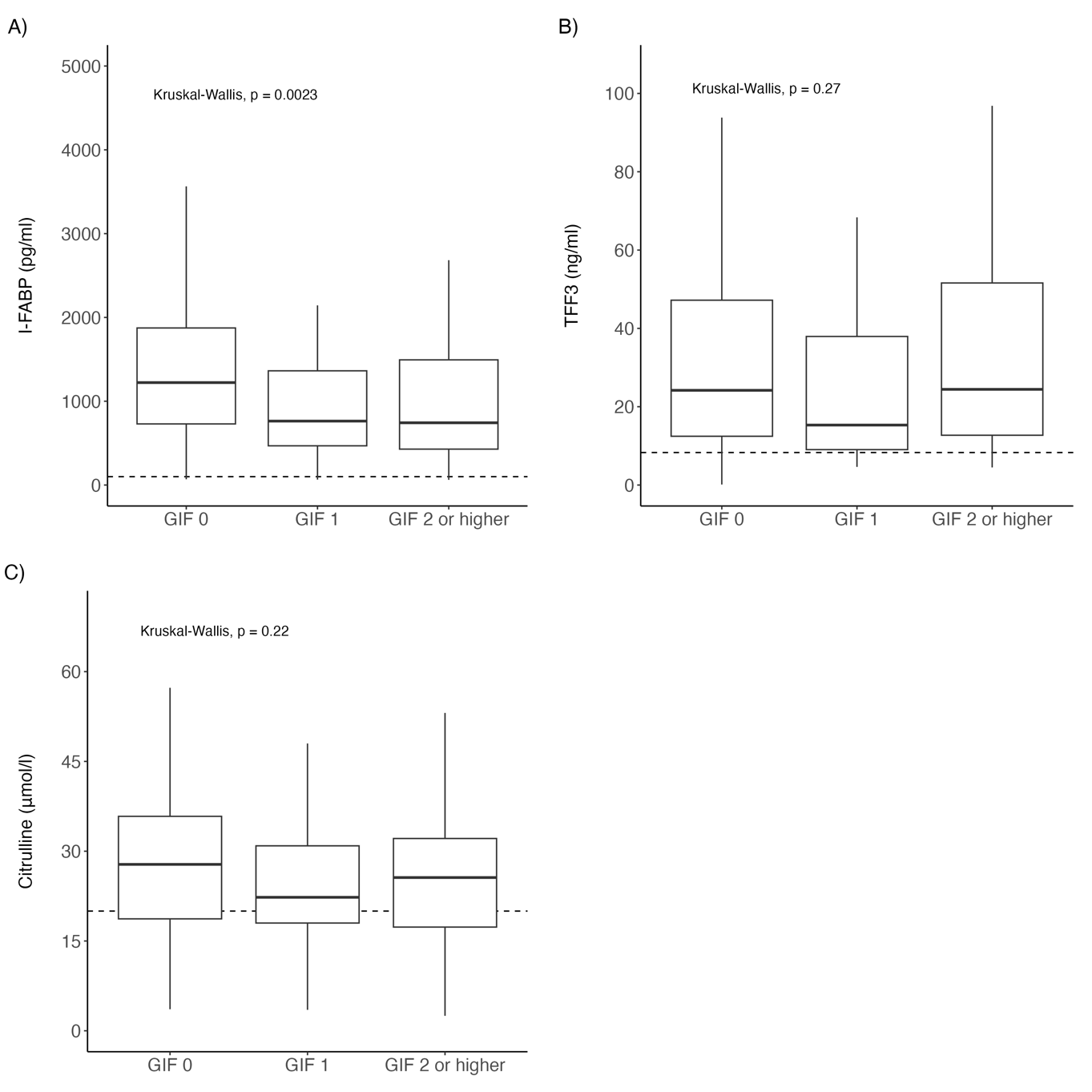


Blood was drawn from patients in the 48 hours preceding a bacteremia event. Plasma levels of (i) I-FABP, ii) TFF3 and iii) citrulline did not significantly differ between patients with Gastrointestinal Failure score 0, 1 or 2 or higher. Abbreviations: I-FABP, intestinal fatty acid-binding protein; TFF3, trefoil factor-3. The dashed line depicts the threshold for abnormal values.

**Additional Fig 4. Plasma biomarkers within subgroups from stratified analyses**
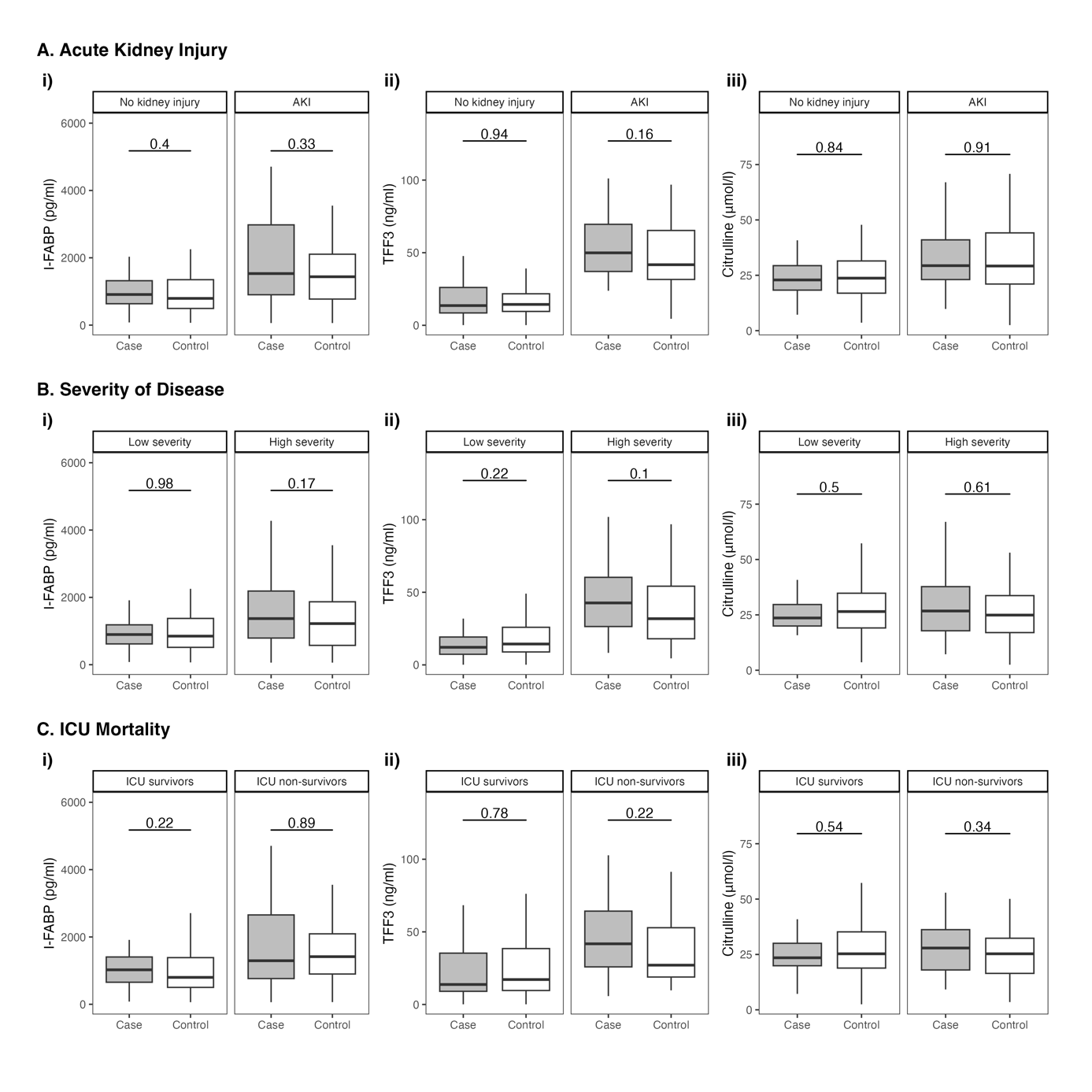
Blood was drawn from patients in the 48 hours preceding a bacteremia event. In stratified analyses for A) presence of acute kidney injury, B) severity of disease, or C) ICU mortality, plasma levels of (i) I-FABP, ii) TFF3 and iii) citrulline did not significantly differ between patients with enterococcal bacteremia as compared to patients with CoNS bacteremia. Acute kidney injury was defined as a RIFLE-score of 2 or higher as present (or measured) in the 48-hour time window immediately preceding the blood culture draw that (later) yielded a first positive result. High severity of disease was defined as Sequential Organ Failure Assessment- score of 7 or higher. Abbreviations: I-FABP, intestinal fatty acid-binding protein; TFF3, trefoil factor-3.
